# *rol-6* and *dpy-10* *C. elegans* mutants have normal mitochondrial function after normalizing to delayed development

**DOI:** 10.17912/micropub.biology.000798

**Published:** 2023-05-03

**Authors:** A. Clare Sparling, Dillon E. King, Joel N. Meyer

**Affiliations:** 1 Nicholas School of Environment, Duke University, Durham NC

## Abstract

Collagen mutations are commonly used in the creation of *Caenorhabditis elegans* transgenic strains, but their secondary effects are not fully characterized *.* We compared the mitochondrial function of N2, *dpy-10, rol-6,* and PE255 *C. elegans* . N2 worms exhibited ~2-fold greater volume, mitochondrial DNA copy number, and nuclear DNA copy number than collagen mutants (p<0.05). Whole-worm respirometry and ATP levels were higher in N2 worms, but differences in respirometry largely disappeared after normalization to mitochondrial DNA copy number. This data suggests that *rol-6* and *dpy-10* mutants are developmentally delayed but have comparable mitochondrial function to N2 worms once the data is normalized to developmental stage.

**Figure 1. Mitochondrial function of roller and dumpy C. elegans:**
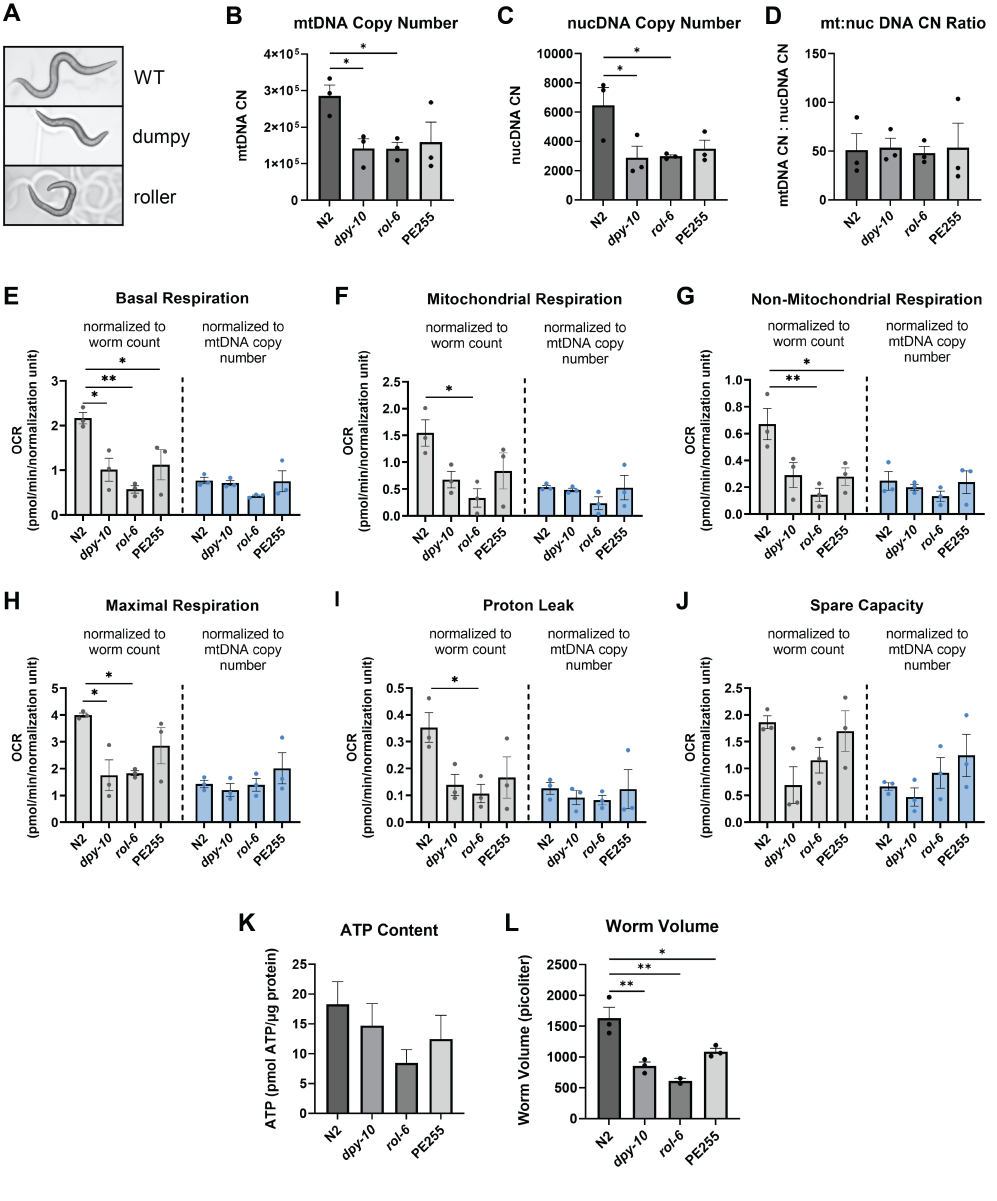
A) Representative images of WT, dumpy, and roller *C. elegans* 48 hours after plating L1s *.* B) Mean mitochondrial DNA copy number across strains. C) Mean nuclear DNA copy number across strains. D) Mean mitochondrial: nuclear DNA copy number (CN) ratio across strains. E) Mean basal respiration across strains. F) Mean mitochondrial respiration across strains. G) Mean non-mitochondrial respiration across strains. H) Mean maximal respiration across strains. I) Mean proton leak across strains. J) Mean spare capacity across strains. K) Mean ATP content across strains. L) Mean worm volume across strains. In all figures, the x-axis represents worm strain. For Figures B-C, copy number is shown on the y-axis. For Figures E-J, OCR in pmol/min, normalized to the appropriate normalization unit, is displayed on the y-axis. Gray bars represent data normalized to worm count. Blue bars represent data normalized to mtDNA CN/10 ^6^ . For Figure K, pmol of ATP, normalized to microgram of protein, is shown on the y-axis. For Figure L, volume in picoliters is displayed on the y-axis. Statistical significance was determined using a one-way ANOVA with a Tukey post hoc test for multiple comparisons (*: p<0.05, **: p<0.01). For Figures E-J, statistical testing was conducted within each normalization method but not between methods. For all graphs, error bars represent the standard error of the mean (SEM).

## Description

When creating new *C. elegans* strains, researchers often include mutated collagen genes in plasmids containing their gene of interest (Fay, 2018) . As the cuticle of *C. elegans* is made of collagen, collagen mutations yield visual phenotypes such as “dumpy” and “roller” that allow researchers to easily screen for *C. elegans* containing the plasmid of interest. “Dumpy” worms are shorter in length, wider, and move slower than the typical laboratory N2 wild-type worms (Fay, 2018) . “Roller” worms are twisted in a right-handed helix, “roll” when they move, and are also slower than N2 worms (Fay, 2018) .

Given the widespread use of strains with dumpy and roller backgrounds in *C. elegans* research, it is important to understand potential secondary effects of collagen mutations. When using a roller strain in previous research, we observed reduced mitochondrial function compared to N2 worms. Mitochondria are organelles involved in many key cellular processes such as energy metabolism, biosynthesis, stress responses, Ca ^2+^ homeostasis, apoptosis, and immune regulation, among others (Spinelli & Haigis, 2018) , (Wang & Youle, 2009) , (Gigori et al., 2018). Thus, if roller or dumpy worms have altered mitochondrial function and a researcher’s area of study could be impacted by mitochondrial function, it should be a consideration in strain design and data interpretation. However, whether collagen mutations are associated with altered mitochondrial function has not been explored.

Here we compared the mitochondrial function of *rol-6 , dpy-10 ,* and PE255 *C. elegans* to the typical laboratory wildtype N2 strain. *rol-6* and *dpy-10* both encode collagen proteins (Kramer et al., 1990) , (Levy et al., 1993) . PE255 worms are *rol-6* mutants with a GFP tag on *sur-5p* and are used to study whole-worm ATP levels (Luz et al., 2016) . However, silencing of the *rol-6* transgene is common in PE255 worms, and thus not all the worms in the PE255 population are rollers. Mitochondrial DNA (mtDNA) and nuclear DNA (nucDNA) copy number, whole worm respirometry, and ATP levels were used to study mitochondrial function. Measurements were made 48 hours after plating L1s that were age-synchronized by overnight hatch following egg isolation, corresponding to the L4 stage in the N2 strain.

First, mtDNA and nucDNA copy number (CN) were assessed across the different strains. N2 *C. elegans* exhibited ~2-fold greater mtDNA and nucDNA CN than all collagen mutants ( Figure 1B -C). However, the ratio of mtDNA CN to nucDNA CN was similar across strains ( Figure 1D ).

Next, whole worm respirometry was assessed. Initial data, normalized to worm count, demonstrated that the N2 worms had a consistently greater oxygen consumption rate (OCR) than the *rol-6* , *dpy-10* , and PE255 strains across the different mitochondrial parameters. However, these differences largely disappeared after normalizing the data to mtDNA CN ( Figure 1E -J). Basal respiration is a measure of the OCR of a resting worm, mitochondrial respiration refers to the OCR associated with the electron transport chain specifically, and non-mitochondrial respiration refers to the difference between basal and mitochondrial respiration. Maximal respiration is an indicator of the OCR of which the organism is capable. Finally, proton leak is a measure of the non-ATP linked respiration, and spare capacity refers to the ability to increase mitochondrial function if needed. No statistically significant differences were found between the strains once the data was normalized to mtDNA CN, although *rol-6* worms appear to have slightly lower basal, mitochondrial, and non-mitochondrial respiration than N2 worms (Figures 1E-J).

Whole-worm ATP levels were then measured. Although the collagen mutants appeared to show lower average ATP levels, none of the differences across strains were statistically significant. N2 worms had an average of 18.28 pmol ATP/µg of protein while *rol-6* , *dpy-10 ,* and PE255 worms had an average of 8.47, 14.72, 12.46 pmol ATP/µg of protein, respectively ( Figure 1K ).

Large differences in nucDNA CN and differences in whole worm respirometry that disappeared after normalization to mtDNA CN suggest that the collagen mutant worms may be developmentally delayed. While not statistically significant, the apparent trend in ATP levels further supports this possibility given that worms’ energetic demands increase between the L3, L4, and adult stages due to germline development (Pazdernik & Schedl, 2013) (Leung et al., 2013) . Thus, a developmentally delayed worm is expected to have lower ATP content when compared to a time-matched L4 worm as was observed when comparing the collagen mutant strains to N2 worms.

Conventional methods for determining worm stage, based on vulval development (Mok et al., 2015) , could not be used due to altered vulval morphology in roller and dumpy worms. Instead, worm volume and the time until first offspring were measured as proxies for developmental stage. The volume of N2 worms was significantly greater than that of *dpy-10* , *rol-6* , and PE255 worms (p<0.01, 0.01, and 0.05, respectively; Figure 1L ). Further, 48 hours after plating synchronized L1s, ten worms of each strain were moved to a new plate. After 72 hours, F1 offspring were observed in the N2 and PE255 populations. However, we did not observe hatching of the F1 generation until 96 hours in the *dpy-10* and *rol-6* populations.

Together, the volume and time to offspring data suggests that the *dpy-10* and *rol-6* worms take longer to mature and thus are developmentally delayed compared to N2 worms. The question then arises of what is driving this delay. It is possible that roller and dumpy worms face greater difficulty in moving to consume food, and this reduction in food intake may drive the observed delay. However, future research would be needed to determine whether this developmental delay is caused by reduced movement, reduced food intake, a secondary genetic effect, or some other driver.

Together, this research does not demonstrate robust differences in mitochondrial function between the collagen mutants and N2 worms, suggesting that these mutants can be used to study questions regarding mitochondrial function. However, developmental stage, rather than age measured by hours of development, should be used to compare strains, given that our results also indicate that the *rol-6* and *dpy-10* mutants are likely developmentally delayed. If this delay is not taken into account by normalizing to mtDNA CN, large differences in mitochondrial function may be apparent and skew data interpretation. To our knowledge, the current literature does not report developmental delays in *rol-6* and *dpy-10* mutants. Further, reports of developmental delays in collagen mutants more broadly are lacking except for a reference to a developmental delay in *sqt-2* worms in a *col-182* background (Noble et al., 2020) and *dpy-1* and *dpy-5* worms (Nyaanga et al., 2022) . As collagen mutant worms are commonly used by *C. elegans* researchers, the developmental delay exhibited by *rol-6* and *dpy-10* strains, and potentially other collagen mutants, should be a consideration for future strain choice and experimental design.

## Methods

Worm Strains and Maintenance:

The following *C. elegans* strains were used: N2 (wildtype), *dpy-10* ( e128 ) *,* PE255 , and *rol-6* ( su1006 ). All strains were obtained from the *Caenorhabditis* Genetics Center, University of Minnesota. Strains were grown at 20°C on K-agar plates seeded with *E. coli* OP50 .

Egg isolation and *C. elegans* Synchronization

Gravid adults were treated with hypochlorite/NaOH to harvest eggs and create a synchronized population of L1 worms for each experiment (Lewis & Fleming, 1995) . Embryos hatched for 16 hours in K+ medium, K-medium supplemented with cholesterol (Boyd et al., 2012) . L1 larvae were then plated on K-agar OP50 *E. coli* plates at 20°C and allowed to grow for 48 hours until the N2 worms reached the L4 stage. Worms forty-eight hours post-plating were used for all experiments.

ATP Quantification

CellTiter-Glo Luminescent Cell Viability Assay (Promega G7572) was used to measure ATP levels. The data was normalized to protein content which was determined using the Pierce bicinchoninic acid assay (Thermo Scientific, Rockford, IL), (Palikaras & Tavernarakis, 2016) . Two hundred worms were flash frozen and stored at -80°C. During analysis, samples were boiled for 15 minutes at 95°C. Cellular debris was then removed by centrifugation. Worm extract was aliquoted and used for ATP quantification and total protein determination.

mtDNA Copy Number Analysis

Six worms from each strain were picked into 90 µL of lysis buffer (25 mM Tricine, pH 8; 80 mM potassium acetate; 11% w/v glycerol; 2.25% v/v DMSO, 1 mg/mL proteinase K in nuclease-free water), which was then flash frozen and stored at -80°C. For analysis, samples were lysed at 65 °C for 1 hour. Two microliters of lysate were used with Power Sybr Green PCR Master Mix (ThermoFisher Scientific, Waltham, MA) for the template in real-time PCR experiments (Leuthner et al., 2021) . Copy number was derived from CT values by using a standard curve based on the pCR 2.1 plasmid which contains the species-specific mitochondrial *nduo-1* gene fragment for mtDNA or *cox-4* gene fragment for nuclear copy number count.

Seahorse Respiration Assays:

20 synchronized worms were placed into each well of a Seahorse XFe96 Extracellular Flux Analyzer microplate, as previously described (Luz et al. 2015) . A modified version of the “mitochondrial stress test” was conducted. Basal oxygen consumption rate (OCR) was measured before injection of 20 µM (final) N,N-dicyclohexylcarbodiimide (DCCD, an ATP synthase inhibitor) to measure ATP-linked respiration or 25 μM (final) carbonyl cyanide 4-(trifluoromethoxy)phenylhydrazone (FCCP, a mitochondrial uncoupler) to determine maximal respiration. Measurements were taken after either FCCP or DCCD injection. 10mM sodium azide, which completely inhibits mitochondrial respiration, was then injected to measure non-mitochondrial OCR. The assay yielded data on Basal OCR, Non-mitochondrial OCR, Maximal OCR, Mitochondrial OCR (Basal OCR – Non-mitochondrial OCR), ATP-linked OCR (Basal OCR – DCCD-inhibited OCR), Spare Capacity (Maximal OCR – Basal OCR), and Proton Leak (DCCD-inhibited OCR – Non-mitochondrial OCR). Each Seahorse experiment included at least five wells per treatment group. Three experimental replicates were conducted per strain. Data was normalized to worm count or mtDNA CN.

Worm Volume:

Worms were washed with K-medium, plated on K-agar plates lacking peptone, and imaged using a Keyence BZX-7100. The WormSizer plugin (Moore et al., 2013) for FIJI was run on the images and used to calculate worm volume. At least 50 worms were analyzed for each replicate.

Statistical Analyses:

GraphPad Prism 9.0 was used for statistical analysis. Mean Seahorse XFe96 Extracellular Flux Analyzer parameters, ATP concentrations, copy number, and worm volume were analyzed with a one-way ANOVA with Tukey’s HSD post-hoc test for multiple comparisons. Significance was determined by p<0.05.

## Reagents

Strain Table:

**Table:** 

Strain	Genotype	Available from
N2	wild type	CGC
HE1006	*rol-6* ( su1006 ) II	CGC
CB128	*dpy-10* ( e128 ) II	CGC
PE255	*feIs5* [ *sur-5p* ::luciferase::GFP + *rol-6* ( su1006 )]	CGC

Copy Number Primers:

**Table:** 

**Genome**	**Forward Primer**	**Reverse Primer**	** T _Anneal_ (°C) **	**Gene**	**Amplicon Size (bp)**
Mitochondrial	5’-AGC GTC ATT TAT TGG GAA GAA GAC-3’	5’-AAG CTT GTG CTA ATC CCA TAA ATG T-3’	60	*nduo-1*	75
Nuclear	5’-GCC GAC TGG AAG AAC TTG TC-3’	5’-GCG GAG ATC ACC TTC CAG TA-3’	60	*cox-4*	164
